# Supplementary material for: Pan-cancer genomic analysis shows hemizygous PTEN loss tumors are associated with immune evasion and poor outcome
Source: Sci Rep. 2023 Mar 28;13:5049. doi: 10.1038/s41598-023-31759-6 (PMC10050165; doi:10.1038/s41598-023-31759-6)
Supplement: Supplementary file 1 — Supplementary Figures. [file 41598_2023_31759_MOESM1_ESM.pdf]

Supplementary Figure 1

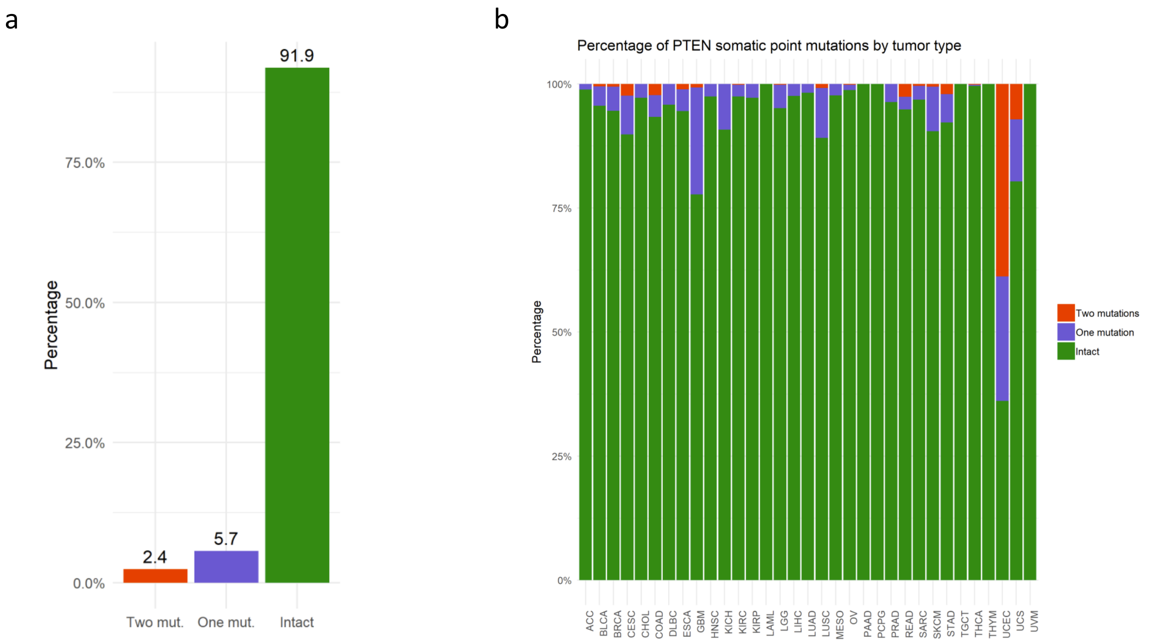

Supplementary Figure 1. *PTEN* somatic mutation data from TCGA. (A) PTEN somatic mutation in PanCancer cohort, b) *PTEN* somatic mutations by tumor type. Plotted using *ggplot2* (https://ggplot2.tidyverse.org/).

Supplementary Figure 2

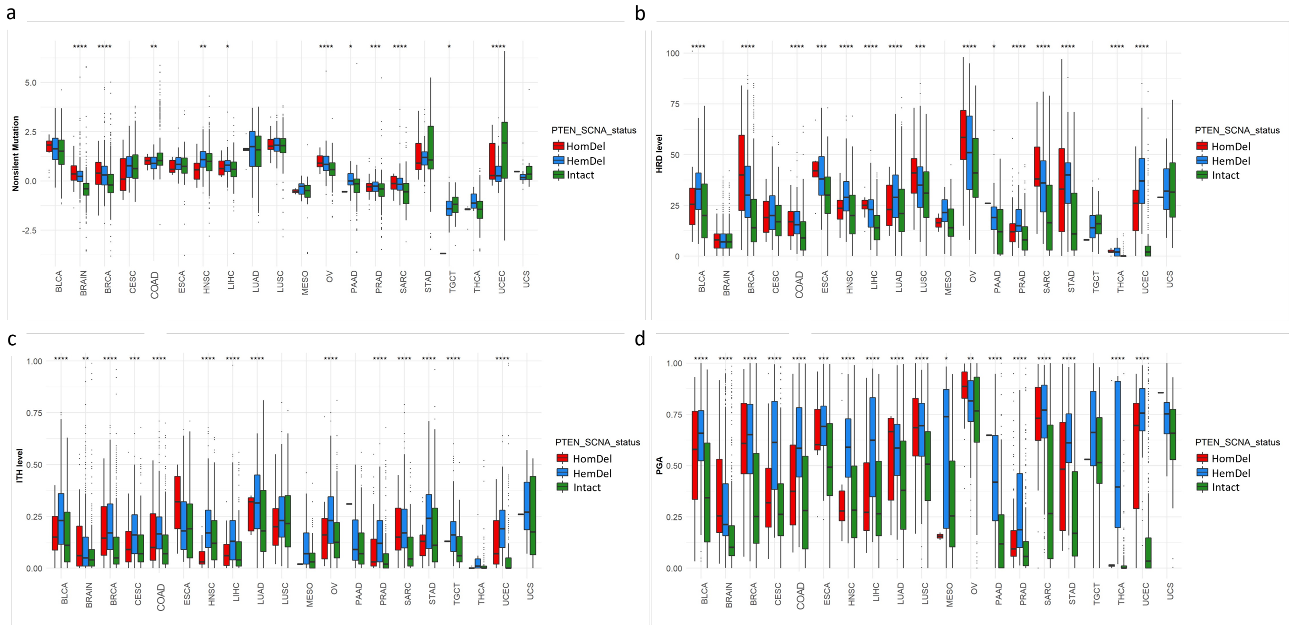

Supplementary Figure 2. *PTEN* deletions are associated with genomic alterations in each tumor type. (**a**) Nonsilent mutations. (**b**) Intratumor heterogeneity. (**c**) Homologous repair defects. (**d**) Percent genome altered. Plotted using *ggplot2* (https://ggplot2.tidyverse.org/).

Supplementary Figure 3

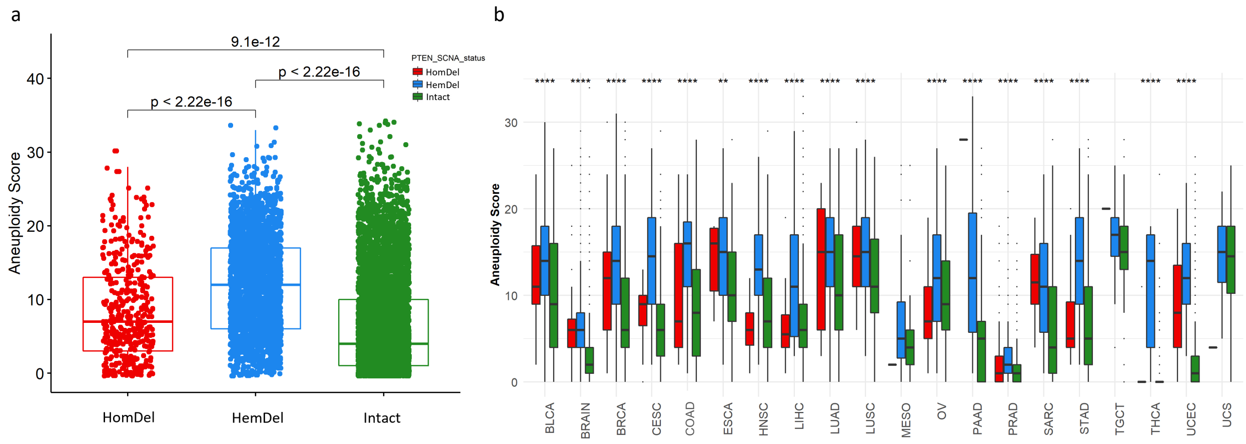

Supplementary Figure 3. Association between aneuploidy levels and *PTEN* inactivation status. (**a**) *PTEN* inactivation is significantly associated with increased levels of aneuploidy. (**b**) Aneuploidy score stratified by tumor type. Prostate and brain tumors showed the lowest levels of aneuploidy across tumor types. Aneuploidy levels were determined based on the length of copy number alterations and normalized by tumor type. The scores range from 0, which indicates low levels of chromosome aberrations (copy number gains and losses). On the other hand, high levels of aneuploidy indicate that the genome of these tumors is highly altered, with larger chromosome gains and losses occurring. Plotted using *ggplot2* (https://ggplot2.tidyverse.org/).

Supplementary Figure 4

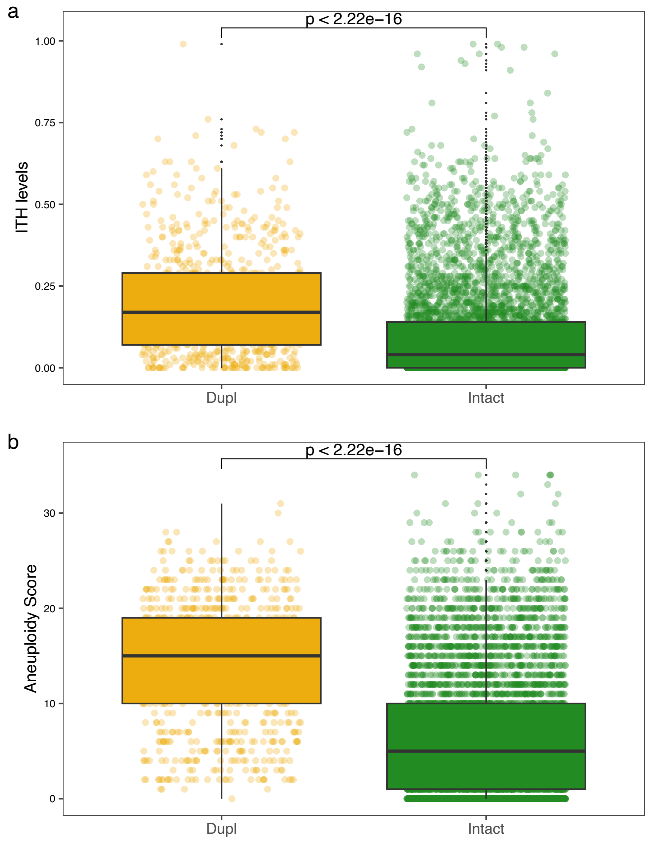

Supplementary Figure 4. *PTEN* duplication is associated with genomic alterations. (**a**) *PTEN* Duplication is associated with elevated levels of Intratumor heterogeneity and (**b**) Aneuploidy score. Aneuploidy levels were determined based on the length of copy number alterations and normalized by tumor type. The scores range from 0, which indicates low levels of chromosome aberrations (copy number gains and losses). On the other hand, high levels of aneuploidy indicate that the genome of these tumors is highly altered, with larger chromosome gains and losses occurring. Plotted using *ggplot2* (https://ggplot2.tidyverse.org/).

Supplementary Figure 5

a

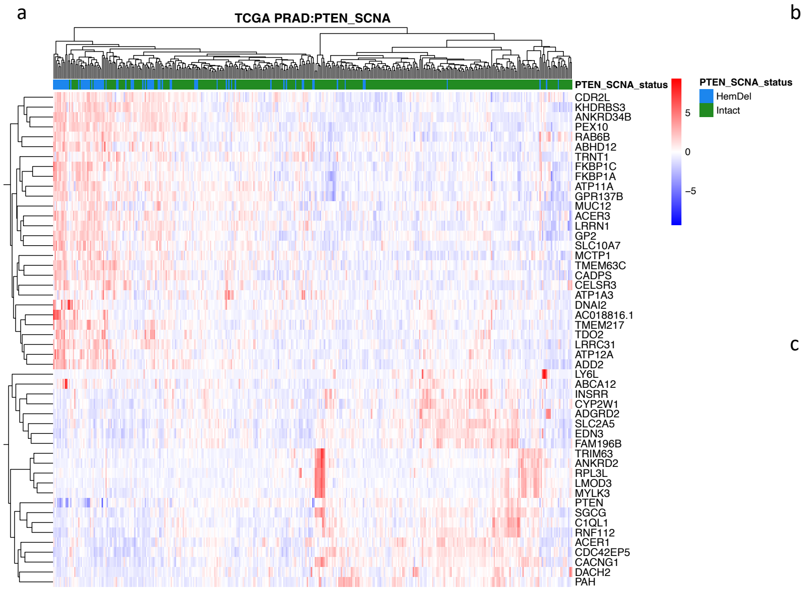

b

ID	Description	qvalue
GO:0007018	microtubule-based movement	3,41E+11
GO:0007059	chromosome segregation	3,41E+11
GO:0000070	mitotic sister chromatid segregation	3,41E+11
GO:0098813	nuclear chromosome segregation	0.0011
GO:1902850	microtubule cytoskeleton organization involved in mitosis	0.0015
GO:0034329	cell junction assembly	0.0018
GO:0050808	synapse organization	0.0028
GO:0032886	regulation of microtubule-based process	0.0038
GO:0000280	nuclear division	0.0065
GO:0007156	homophilic cell adhesion via plasma membrane adhesion molecules	0.0072

c

ID	Description	qvalue
GO:0002181	cytoplasmic translation	3,03E+07
GO:0006936	muscle contraction	1,28E+10
GO:0016054	organic acid catabolic process	1,86E+11
GO:0044282	small molecule catabolic process	8,90E+11
GO:0008015	blood circulation	0.0020
GO:0086005	ventricular cardiac muscle cell action potential	0.0070
GO:0010273	detoxification of copper ion	0.0154
GO:1990169	stress response to copper ion	0.0154
GO:0061687	detoxification of inorganic compound	0.0174
GO:0086009	membrane repolarization	0.0174

Supplementary Figure 5. *PTEN* HemDel PRAD showed distinct transcriptome activity. (**a**) Transcriptome heatmap exhibiting clustering of top 50 DEGs (Log2Fold change >0.58). (**b**) Enriched GO (BP) pathways of the upregulated genes. (**c**) Enriched GO (BP) pathways of the downregulated genes. The color scale in the heatmap represents the Z-score of the normalized read counts for each gene, where the red scale color indicates upregulated and blue low-expressed genes. Enrichment analyses were performed using *clusterProfiler* and *p*-adjusted value = 0.05 as the cutoff. Tables show the top 10 most significant Gene Ontology (BP) terms. Plotted using *pheatmap* (https://rdocumentation.org/packages/pheatmap/versions/1.0.12).

Supplementary Figure 6

a

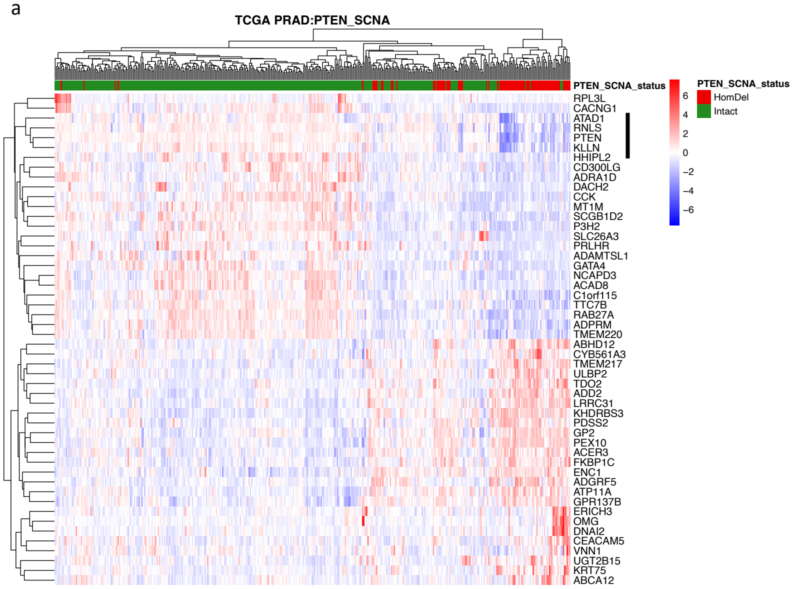

b

ID	Description	qvalue
GO:0044770	cell cycle phase transition	1,09E+12
GO:0007059	chromosome segregation	1,39E+12
GO:0007018	microtubule-based movement	2,92E+11
GO:1901987	regulation of cell cycle phase transition	4,63E+12
GO:0006281	DNA repair	6,03E+11
GO:0000819	sister chromatid segregation	0.0017
GO:0000070	mitotic sister chromatid segregation	0.0017
GO:0044782	cilium organization	0.0017
GO:0001578	microtubule bundle formation	0.0020
GO:0048285	organelle fission	0.0033

c

ID	Description	qvalue
GO:0008015	blood circulation	8,75E+09
GO:0006936	muscle contraction	1,16E+11
GO:0003018	vascular process in circulatory system	3,47E+11
GO:0034765	regulation of ion transmembrane transport	4,05E+11
GO:0099177	regulation of trans-synaptic signaling	0.0011
GO:0015844	monoamine transport	0.0022
GO:0008016	regulation of heart contraction	0.0038
GO:0016054	organic acid catabolic process	0.0041
GO:0072503	cellular divalent inorganic cation homeostasis	0.0041
GO:0007188	adenylate cyclase-modulating G protein-coupled receptor signaling pathway	0.0041

Supplementary Figure 6. *PTEN* HomDel PRAD showed distinct transcriptome activity. **a)** Transcriptome heatmap exhibiting clustering of top 50 DEGs (Log2Fold change >0.58). **b)** Enriched GO (BP) pathways of the upregulated genes. **c)** Enriched GO (BP) pathways of the downregulated genes. The color scale in the heatmap represents the Z-score of the normalized read counts for each gene, where the red scale color indicates upregulated and blue low-expressed genes. Enrichment analyses were performed using *clusterProfiler* and *p*-adjusted value = 0.05 as the cutoff. Tables show the top 10 most significant Gene Ontology (BP) terms. Vertical bar indicates the closely linked passenger genes (*ATAD1, RNLS* and *KLLN*) that are concurrently down-regulated when *PTEN* is lost by a large deletion of chromosome 10. Plotted using *pheatmap* (https://rdocumentation.org/packages/pheatmap/versions/1.0.12).

Supplementary Figure 7

a

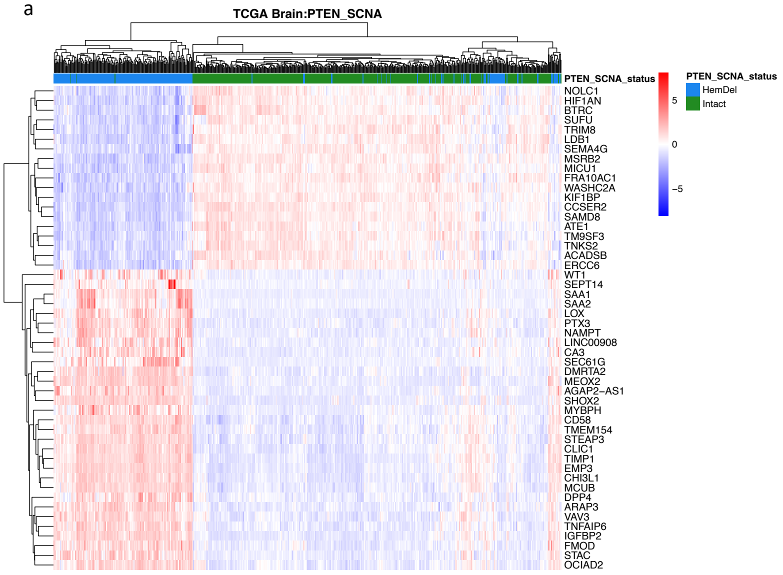

b

ID	Description	qvalue
GO:0002250	adaptive immune response	9,17E-10
GO:0042110	T cell activation	3,96E-06
GO:0030198	extracellular matrix organization	5,64E-05
GO:0002443	leukocyte mediated immunity	2,69E-01
GO:0002237	response to molecule of bacterial origin	4,85E-01
GO:0045785	positive regulation of cell adhesion	6,54E-02
GO:1903037	regulation of leukocyte cell-cell adhesion	7,12E-02
GO:0007159	leukocyte cell-cell adhesion	1,31E+00
GO:0050900	leukocyte migration	1,39E-01
GO:0050863	regulation of T cell activation	2,64E+00

c

ID	Description	qvalue
GO:0022613	ribonucleoprotein complex biogenesis	1,67E+08
GO:0008380	RNA splicing	1,67E+08
GO:0050804	modulation of chemical synaptic transmission	2,70E+10
GO:0099003	vesicle-mediated transport in synapse	6,22E+09
GO:0022618	ribonucleoprotein complex assembly	8,34E+09
GO:0071826	ribonucleoprotein complex subunit organization	1,97E+11
GO:0050808	synapse organization	5,09E+10
GO:0016197	endosomal transport	5,10E+11
GO:0043543	protein acylation	7,89E+10
GO:0001505	regulation of neurotransmitter levels	8,50E+11

Supplementary Figure 7. *PTEN* HemDel Brain (GBM + LGG) showed distinct transcriptome activity. (**a**) Transcriptome heatmap exhibiting clustering of top 50 DEGs (Log2Fold change >0.58). (**b**) Enriched GO (BP) pathways of the upregulated genes. (**c**) Enriched GO (BP) pathways of the downregulated genes. The color scale in the heatmap represents the Z-score of the normalized read counts for each gene, where the red scale color indicates upregulated and blue low-expressed genes. Enrichment analyses were performed using *clusterProfiler* and *p*-adjusted value = 0.05 as the cutoff. Tables show the top 10 most significant Gene Ontology (BP) terms. Plotted using *pheatmap* (https://rdocumentation.org/packages/pheatmap/versions/1.0.12).

Supplementary Figure 8

a

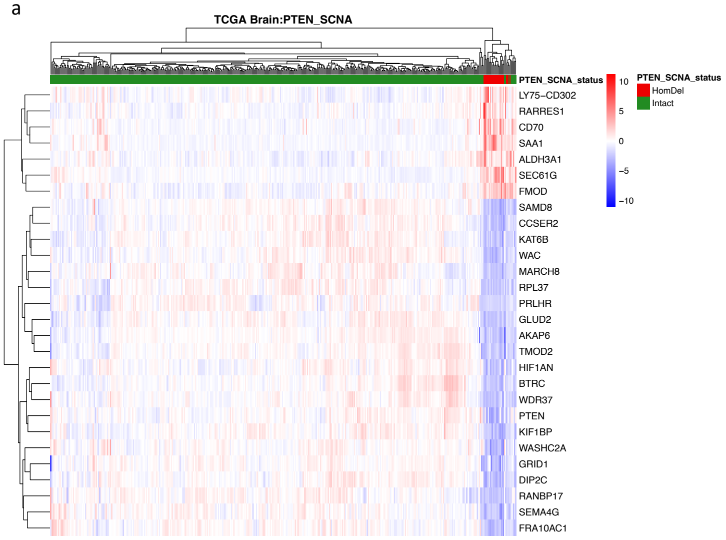

b

ID	Description	qvalue
GO:0002250	adaptive immune response	4,71E-13
GO:0042110	T cell activation	2,76E-09
GO:0050867	positive regulation of cell activation	3,49E-08
GO:0002443	leukocyte mediated immunity	7,82E-08
GO:1903037	regulation of leukocyte cell-cell adhesion	2,31E-06
GO:0050778	positive regulation of immune response	3,60E-05
GO:0007159	leukocyte cell-cell adhesion	7,56E-06
GO:0019221	cytokine-mediated signaling pathway	7,58E-06
GO:0050870	positive regulation of T cell activation	1,71E-02
GO:0001819	positive regulation of cytokine production	1,10E-01

c

ID	Description	qvalue
GO:0050804	modulation of chemical synaptic transmission	1,60E+01
GO:0099177	regulation of trans-synaptic signaling	1,60E+01
GO:0099504	synaptic vesicle cycle	2,43E+03
GO:0050808	synapse organization	9,90E+04
GO:0006397	mRNA processing	2,35E+08
GO:0016079	synaptic vesicle exocytosis	2,35E+08
GO:0060078	regulation of postsynaptic membrane potential	7,11E+07
GO:0099643	signal release from synapse	1,55E+09
GO:0016358	dendrite development	5,48E+08
GO:0001505	regulation of neurotransmitter levels	1,31E+09

Supplementary Figure 8. *PTEN* HomDel Brain (GBM + LGG) showed distinct transcriptome activity. (**a**) Transcriptome heatmap exhibiting clustering of top 50 DEGs (Log2Fold change >0.58). Of the 4106 overexpressed genes in the HomDel tumors 66.7% were also overexpressed in the 3562 HemDel tumors. Similarly, of the 7332 underexpressed genes in the HomDel tumors 59.9% were also underexpressed in the 10902 underexpressed genes in the HemDel tumors (**b**) Enriched GO (BP) pathways of the upregulated genes. The color scale in the heatmap represents the Z-score of the normalized read counts for each gene, where the red scale color indicates upregulated and blue low-expressed genes. Enrichment analyses were performed using *clusterProfiler* and *p*-adjusted value = 0.05 as the cutoff. Tables show the top 10 most significant Gene Ontology (BP) terms. Plotted using *pheatmap* (https://rdocumentation.org/packages/pheatmap/versions/1.0.12).

Supplementary Figure 9

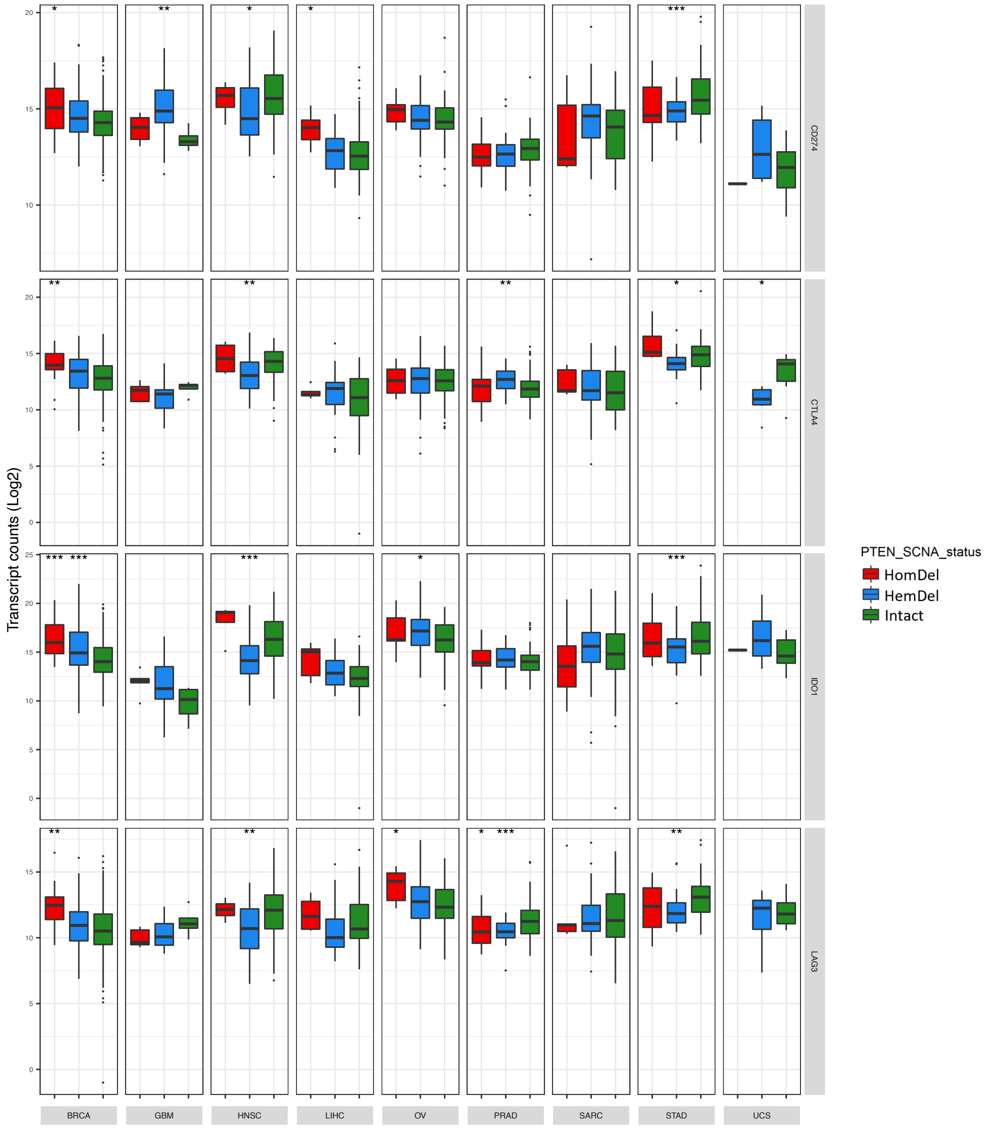

Supplementary Figure 9. The RNA-sequencing expression level of immunomodulatory genes *CD274* (*PD-L1*), *CTLA4*, *IDO1*, and *LAG3* in BRCA, GBM, HNSC, LIHC, OV, PRAD, SARC, STAD, and UCS. *: *p*-value < 0.05; **: *p*-value < 0.01, by Mann-Whitney test. *CD274* (*PD-L1*), Programmed Cell Death 1 Ligand 1; *CTLA4*, Cytotoxic T-Lymphocyte Associated Protein 4; *IDO1*, Indoleamine 2,3-Dioxygenase 1; LAG3, Lymphocyte Activating 3. Plotted using *ggplot2* (https://ggplot2.tidyverse.org/).

Supplementary Figure 10

a

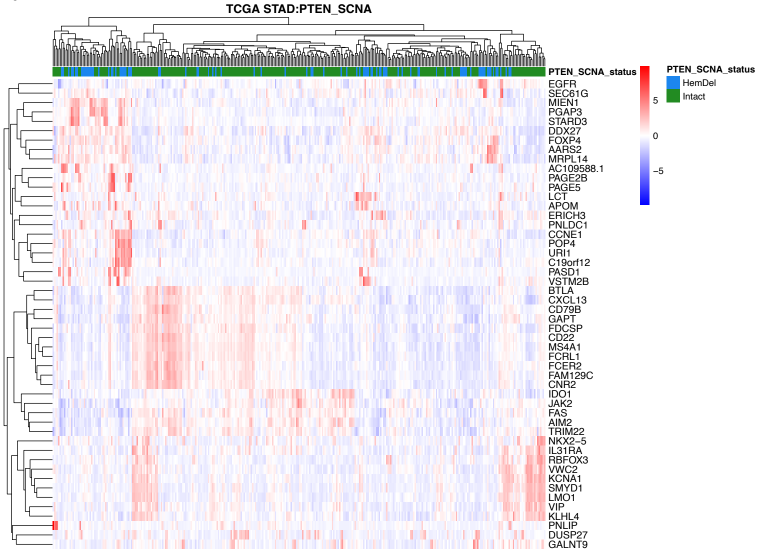

b

ID	Description	qvalue
GO:0000819	sister chromatid segregation	1,75E+06
GO:0000070	mitotic sister chromatid segregation	1,75E+06
GO:0007059	chromosome segregation	2,77E+07
GO:0000280	nuclear division	2,77E+07
GO:0098813	nuclear chromosome segregation	5,81E+06
GO:0051321	meiotic cell cycle	1,05E+12
GO:0016126	sterol biosynthetic process	5,01E+11
GO:0006695	cholesterol biosynthetic process	5,01E+11
GO:1902653	secondary alcohol biosynthetic process	5,01E+11
GO:0034660	ncRNA metabolic process	7,25E+11

c

ID	Description	qvalue
GO:0002250	adaptive immune response	1,55E-22
GO:0051249	regulation of lymphocyte activation	5,34E-12
GO:0042110	T cell activation	7,73E-12
GO:0050778	positive regulation of immune response	9,10E-11
GO:0002443	leukocyte mediated immunity	8,74E-09
GO:0007159	leukocyte cell-cell adhesion	3,17E-07
GO:1903037	regulation of leukocyte cell-cell adhesion	4,46E-07
GO:0050867	positive regulation of cell activation	7,92E-07
GO:0070661	leukocyte proliferation	4,32E-06
GO:0046651	lymphocyte proliferation	1,44E-04

Supplementary Figure 10. *PTEN* HemDel STAD showed distinct transcriptome activity. (**a**) Transcriptome heatmap exhibiting clustering of top 50 DEGs (Log2Fold change >0.58). (**b**) Enriched GO (BP) pathways of the upregulated genes. (**c**) Enriched GO (BP) pathways of the downregulated genes. The color scale in the heatmap represents the Z-score of the normalized read counts for each gene, where the red scale color indicates upregulated and blue low-expressed genes. Enrichment analyses were performed using *clusterProfiler* and *p*-adjusted value = 0.05 as the cutoff. Tables show the top 10 most significant Gene Ontology (BP) terms. Plotted using *pheatmap* (https://rdocumentation.org/packages/pheatmap/versions/1.0.12).

Supplementary Figure 11

a

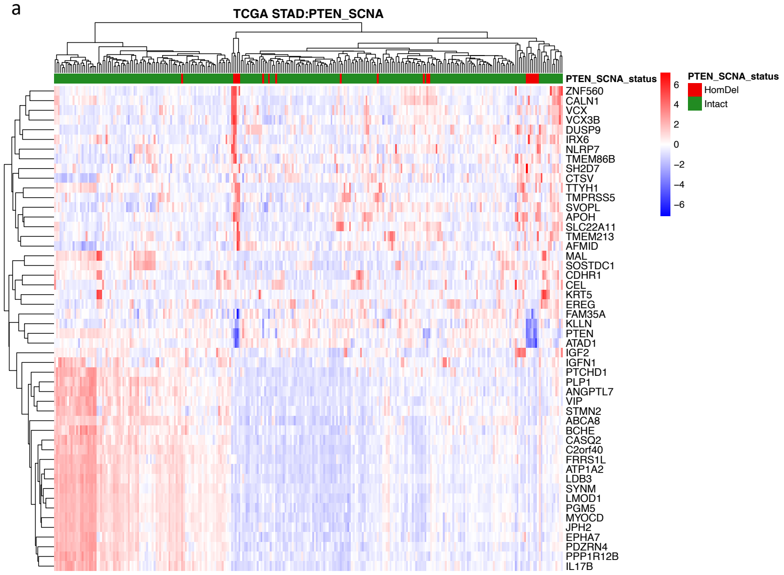

b

ID	Description	qvalue
GO:0003012	muscle system process	5,90E-07
GO:0051480	regulation of cytosolic calcium ion concentration	7,86E+02
GO:0008015	blood circulation	2,35E+04
GO:0010959	regulation of metal ion transport	7,72E+05
GO:0072507	divalent inorganic cation homeostasis	7,96E+05
GO:0008016	regulation of heart contraction	2,15E+08
GO:0042692	muscle cell differentiation	3,11E+07
GO:0060402	calcium ion transport into cytosol	5,62E+08
GO:0034765	regulation of ion transmembrane transport	7,90E+07
GO:0006816	calcium ion transport	1,21E+09

Supplementary Figure 11. *PTEN* HomDel STAD showed distinct transcriptome activity. (**a**) Transcriptome heatmap exhibiting clustering of top 50 DEGs (Log2Fold change >0.58). (**b**) Enriched GO (BP) pathways of the upregulated genes. The color scale in the heatmap represents the Z-score of the normalized read counts for each gene, where the red scale color indicates upregulated and blue low-expressed genes. Enrichment analyses were performed using *clusterProfiler* and *p*-adjusted value = 0.05 as the cutoff. Tables show the top 10 most significant Gene Ontology (BP) terms. Plotted using *pheatmap* (https://rdocumentation.org/packages/pheatmap/versions/1.0.12).

Supplementary Figure 12

a

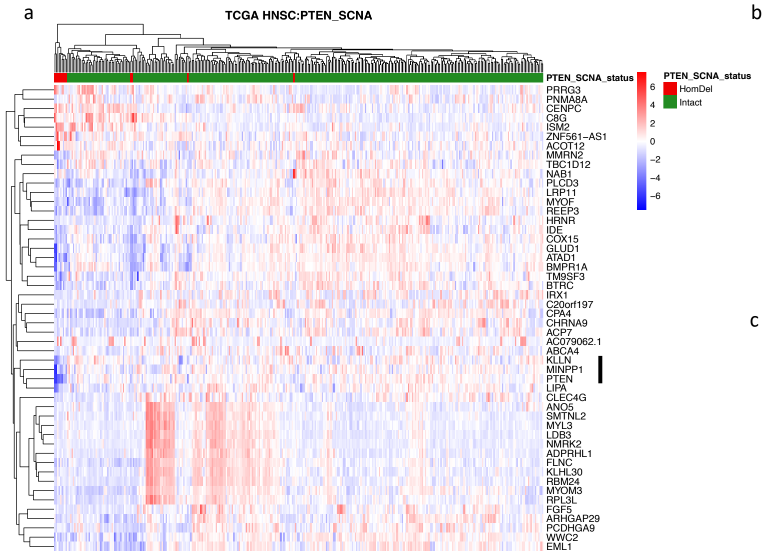

b

ID	Description	qvalue
GO:0006260	DNA replication	8,25E+09
GO:0007059	chromosome segregation	8,25E+09
GO:0051321	meiotic cell cycle	5,49E+09
GO:0098813	nuclear chromosome segregation	1,39E+12
GO:0000280	nuclear division	1,39E+12
GO:0034508	centromere complex assembly	1,92E+11
GO:0006335	DNA replication-dependent nucleosome assembly	0.0023
GO:0034723	DNA replication-dependent nucleosome organization	0.0023
GO:0071824	protein-DNA complex subunit organization	0.0028
GO:0007098	centrosome cycle	0.0028

c

ID	Description	qvalue
GO:0003012	muscle system process	2,71E-09
GO:0055001	muscle cell development	2,11E+00
GO:0014706	striated muscle tissue development	3,43E+01
GO:0030239	myofibril assembly	7,66E+00
GO:0007517	muscle organ development	2,85E+04
GO:0031032	actomyosin structure organization	3,33E+04
GO:0048738	cardiac muscle tissue development	3,80E+04
GO:0050879	multicellular organismal movement	6,46E+04
GO:0050881	musculoskeletal movement	6,46E+04
GO:0008015	blood circulation	3,51E+06

Supplementary Figure 12. *PTEN* HomDel HNSC showed distinct transcriptome activity. (**a**) Transcriptome heatmap exhibiting clustering of top 50 DEGs (Log2Fold change >0.58). (**b**) Enriched GO (BP) pathways of the upregulated genes. (**c**) Enriched GO (BP) pathways of the downregulated genes. The color scale in the heatmap represents the Z-score of the normalized read counts for each gene, where the red scale color indicates upregulated and blue low-expressed genes. Enrichment analyses were performed using *clusterProfiler* and *p*-adjusted value = 0.05 as the cutoff. Tables show the top 10 most significant Gene Ontology (BP) terms. Vertical bar indicates the two closely linked passenger genes (*MINPP1 and KLLN*) that are concurrently down-regulated when PTEN is lost by a large deletion of chromosome 10. Plotted using *pheatmap* (https://rdocumentation.org/packages/pheatmap/versions/1.0.12).

Supplementary Figure 13

a

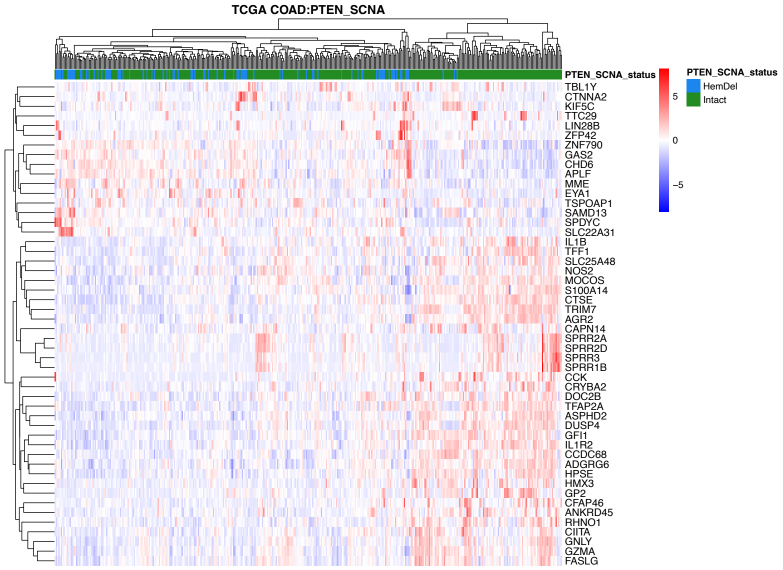

b

ID	Description	qvalue
GO:0034341	response to interferon-gamma	1,93E+10
GO:0002449	lymphocyte mediated immunity	1,93E+10
GO:0002250	adaptive immune response	1,93E+10
GO:0002228	natural killer cell mediated immunity	8,21E+10
GO:0045333	cellular respiration	1,12E+12
GO:0002396	MHC protein complex assembly	2,46E+12
GO:0001906	cell killing	2,49E+12
GO:0009615	response to virus	2,49E+12
GO:0001909	leukocyte mediated cytotoxicity	3,67E+12
GO:0002495	antigen processing and presentation of peptide antigen via MHC class II	3,67E+12

Supplementary Figure 13. *PTEN* HemDel COAD showed distinct transcriptome activity. (**a**) Transcriptome heatmap exhibiting clustering of top 50 DEGs (Log2Fold change >0.58). (**b**) Enriched GO (BP) pathways of the downregulated genes. The color scale in the heatmap represents the Z-score of the normalized read counts for each gene, where the red scale color indicates upregulated and blue low-expressed genes. Enrichment analyses were performed using *clusterProfiler* and *p*-adjusted value = 0.05 as the cutoff. Tables show the top 10 most significant Gene Ontology (BP) terms. Plotted using *pheatmap* (https://rdocumentation.org/packages/pheatmap/versions/1.0.12).

Supplementary Figure 14

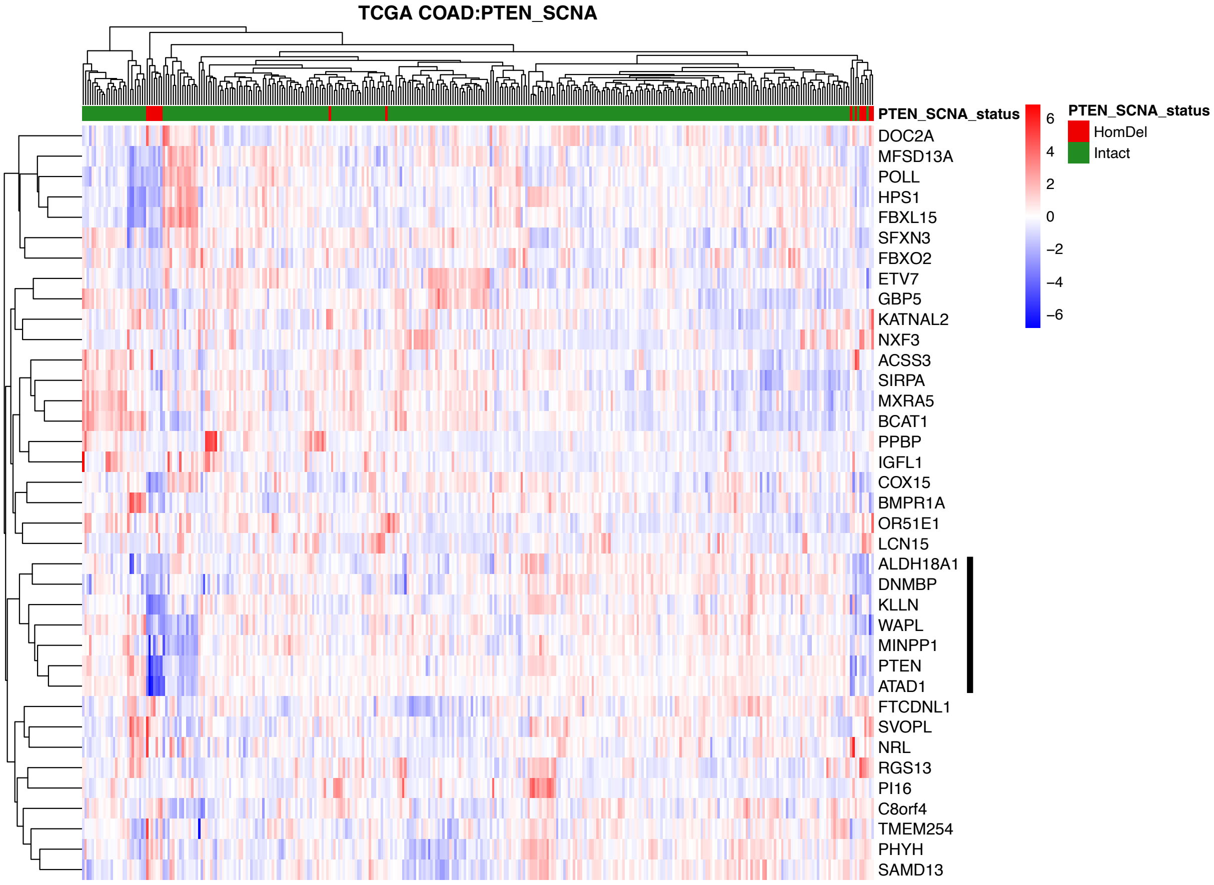

Supplementary Figure 14. *PTEN* HomDel COAD showed distinct transcriptome activity. Transcriptome heatmap exhibiting clustering of top 50 DEGs (Log2Fold change >0.58). Vertical bar indicates the closely linked passenger genes (*ALDH18A1, DNMBP, KLLN*, *WAPL, MINPP1, ATAD1)* that are concurrently down-regulated when PTEN is lost by a large deletion of chromosome 10. Plotted using *pheatmap* (https://rdocumentation.org/packages/pheatmap/versions/1.0.12).

Supplementary Figure 15

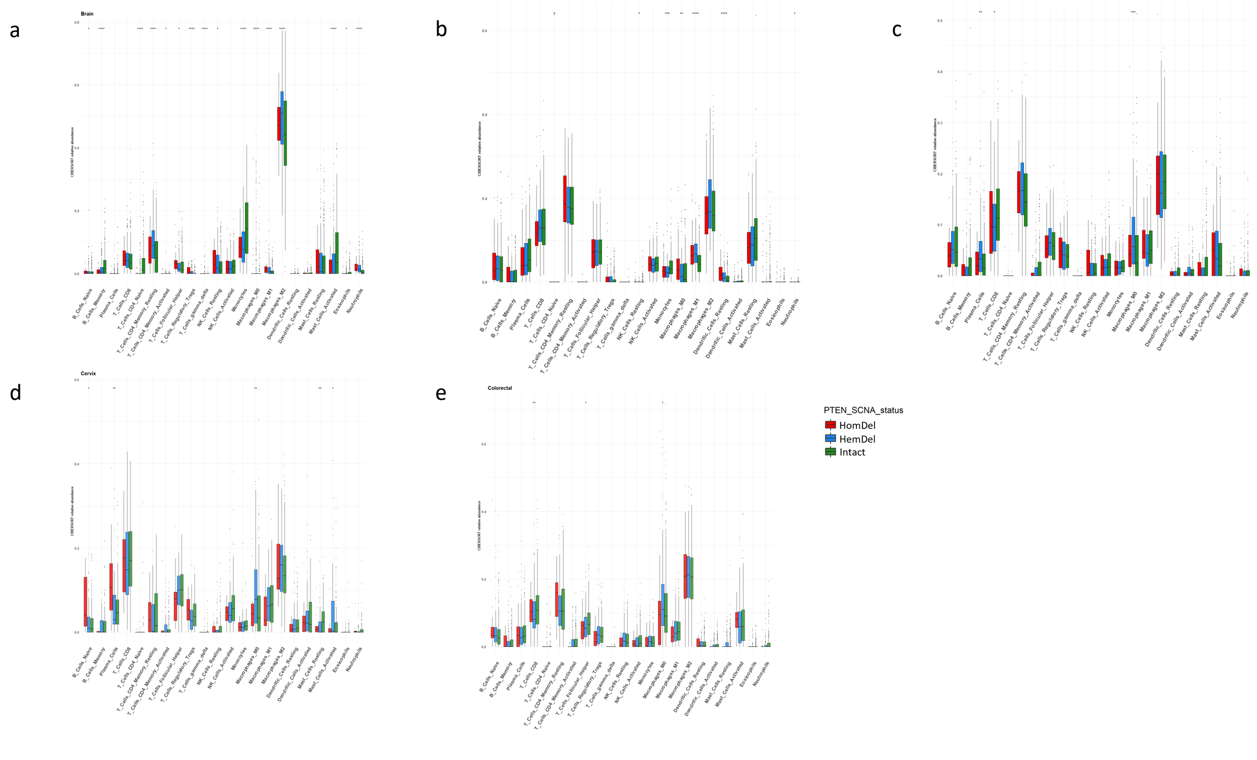

Supplementary Figure 15. CIBERSORT-derived immune-cell abundance of 22 distinct cell subsets based on *PTEN* status and tumor type. Y-axis shows CIBERSORT relative scores. (**a**) *PTEN* HemDel in brain tumors was linked to higher CD4 T memory cells and M2 macrophage abundance and lower M1 macrophages, monocytes, and CD8 T cells. (**b**) Prostate tumors with *PTEN* HemDel showed increased M1 macrophages and decreased M0 macrophages and monocytes. (**c**) Stomach tumors with *PTEN* HemDel showed increased plasma and M0 macrophages and decreased in CD8 T cells. (**d**) Cervical tumors with *PTEN* HemDel were linked to higher M0 macrophages and mast cells activated. (**e**) Colorectal tumors with *PTEN* HemDel were linked to higher M0 macrophages and lower CD8 T cells. *: p-value < 0.05; **: p-value < 0.01, by Mann-Whitney test. DC, dendritic cell; NK, natural-killer cell; PCa, prostate cancer; Treg, regulatory T cell; M0, M0 macrophage; M1, M1 macrophage; M2, M2 macrophage. Plotted using *ggplot2* (https://ggplot2.tidyverse.org/).
