# Supplementary material for: Pan-cancer genomic analysis shows hemizygous PTEN loss tumors are associated with immune evasion and poor outcome
Source: Sci Rep. 2023 Mar 28;13:5049. doi: 10.1038/s41598-023-31759-6 (PMC10050165; doi:10.1038/s41598-023-31759-6)
Supplement: Supplementary file 2 — Supplementary Tables. [file 41598_2023_31759_MOESM2_ESM.pdf]

Supplementary Table 1

PTEN_SCNA_status	TP53 Mut	TP53 WT	χ2	p-value
HemDel	1343	1155	448.49	< 0.001
HomDel	194	223		
Intact	1786	4206		
**Total**	**3323**	**5584**		

Supplementary Table 1. A chi-square test of independence showed that there was a significant association between *PTEN* status and somatic mutation status of *TP53*, $\chi 2$ (2, N = 7866) = 448.4, p = < 0.001. *TP53* mutation status was more likely than *TP53* WT in PTEN HemDel.

Supplementary Table 2

a)

Variable	PanCancer (OS)	
	**HR (95% CI)**	**p***
**PTEN CNA + TP53**		
Intact + WT	-	-
Intact + MonAll	2.07 (1.86 to 2.3)	p<0.001
Intact + BiAll	1.39 (1.06 to 0.0)	0.019
HomDel + WT	2.09 (1.68 to 2.6)	p<0.001
HomDel + MonAll	1.55 (1.19 to 2.02)	0.001
HomDel + BiAll	3.79 (1.7 to 8.46)	0.001
HemDel + WT	2.67 (2.41 to 2.95)	p<0.001
HemDel + MonAll	2.36 (2.12 to 2.63)	p<0.001
HemDel + BiAll	2.84 (2.17 to 3.72)	p<0.001

b)

Variable	HNSC (OS)	
	**HR (95% CI)**	**p***
**PTEN CNA + TP53**		
Intact + WT	-	-
Intact + MonAll	1.90 (1.29 to 2.81)	0.001
Intact + BiAll	1.59 (0.91 to 2.55)	0.10
HomDel + WT	1.6 (0.00 to -)	-
HomDel + MonAll	0.78 (0.10 to 5.7)	0.8
HomDel + BiAll	4.14 (0.57 to 30.2)	0.16
HemDel + WT	1.99 (0.99 to 4.01)	0.053
HemDel + MonAll	1.67 (1.08 to 2.6)	0.023
HemDel + BiAll	1.47 (0.65 to 3.29)	0.35

Supplementary Table 2. Cox Model analysis of overall survivor for the co-occurrence of *TP53* mutation and *PTEN* SCNA. **(A)** *PTEN* HemDel and *TP53* monoallelic mutation tumors are associated with similar hazard ratio levels across the entire TCGA cohort **(B)** Head and neck tumors also showed that *PTEN* HemDel and *TP53* monoallelic mutation exhibited similar hazard ratios. HR, Hazard Ratio; *TP53* MonAll, monoallelic loss for *TP53;* BiAll, biallelic loss for *TP53*.

Supplementary table 3

				DE Genes (HemDel vs Intact)		DE Genes (HomDel vs Intact)	
**Tumor type**	**HemDel**	**HomDel**	**Int**	**Up**	**Down**	**Up**	**Down**
Brain	235	22	406	3562	10902	4106	7332
Colorectal	86	16	319	1086	1536	23	175
Cervix	74	14	186	728	649	743	512
Head and Neck	117	13	329	1727	1842	444	1148
Prostate	73	85	324	2076	2966	3304	3835
Sarcoma	120	15	106	2032	3725	241	708
Stomach	90	20	256	3216	2814	683	1434
	795	185	1926	14427	24432	9544	15144

Supplementary Table 3. The number of differentially expressed genes (DEGs) comparing HemDel and HomDel to intact in seven solid tumors. FDR < 0.05. *PTEN* deletion status: HemDel: *PTEN* hemizygous deletion. HomDel: *PTEN* homozygous deletion. Int: *PTEN* intact. FDR, False discovery rate.
